# Supplementary material for: Dedifferentiation-driven oncogenic stemness promotes tumor-sustaining adaptability in the intestinal epithelium
Source: Cell Death Dis. 2026 Apr 17;17(1):514. doi: 10.1038/s41419-026-08669-2 (PMC13216273; doi:10.1038/s41419-026-08669-2)
Supplement: Supplementary file 1 — Legends for supplementary figures [file 41419_2026_8669_MOESM1_ESM.docx]

**Supplementary figure legend**

**Figure S1. Differential expression of genes in the double mutant villi epithelium (DMV) from the wild-type villi (WTV) epithelium. A,** Bar graph showing the number of differentially expressed genes (DEGs). A total of 2,435 DEGs were detected in the mutant villi epithelium, with 960 being upregulated and 1,475 genes downregulated (left). Unsupervised hierarchical clustering heatmap of the DEGs showing clear segregation of the samples by group (right). B, Heatmap showing differential expression of the transcriptional targets Smad4 and β-catenin and key Lgr5+ stem and lineage markers of the intestinal epithelium.

**Figure S2: Heatmap from GSEA** showing positive enrichment of the signatures of cellular plasticity and growth and in the double mutant villi (DMV) versus wild-type villi (WTV). n =6 (3 replicates per treatment).

**Figure S3. Organoids from the double mutant villi epithelium can grow in the combined absence of Epidermal Growth Factor (E), R-spondin (R), and Noggin (N).** Wild-type crypt epithelium (WT), double-mutant crypt epithelium (CD), or the double-mutant villi (VD) were plated in the presence or the combined absence of the three growth factors (ENR). Unlike the wild-type, which showed no organoids without the ENR, the crypt and villi-derived organoids from the mutant mouse showed growth factor independence seven days after plating.

**Figure S4: Metabolic alterations:** A, Heatmap and GSEA plot showing positive enrichment of the gene signatures in the double mutant villi (DMV) versus wild-type villi (WTV). n = 6 (3 replicates per treatment). B, Densitometry analysis on western blotting: Data were first normalized to the lowest β-actin value, and target protein levels were then normalized to their respective β-actin controls.

**Figure S5. Batch integration analysis:** **A**, dot plot of various intestinal lineage markers expressed in the various clusters from each sample; blue arrows point to the clusters most enriched for Lgr5+ stem cell marker genes. The horizontal boxed region points to the Lgr5^+^ marker gene expression. The size of each dot corresponds to the fraction of cells within a cluster expressing the indicated gene, while the color intensity represents the average expression level, and **B,** the relative number of cells in the various clusters of the three samples. control_adata = wild-type epithelium, v_adata = mutant villi, c_adata = mutant crypt.

**Figure S6. Non-integrated analysis.** Dot plot of various intestinal lineage markers expressed in the various clusters of the wild-type epithelium (**A**), dedifferentiating cells in the double-mutant villi epithelium (**B**), and the double-mutant crypts (**C**). The size of each dot corresponds to the fraction of cells within a cluster expressing the indicated gene, while the color intensity represents the average expression level.

**Original Data Legend.**

Original western blot showing molecular weight markers and densitometry analysis: The corresponding molecular weight on the ladder and the pre-stained ladder used are shown on the left and right, respectively. The far-right and far-left well contains the pre-stained ladder. For densitometric analysis, data were first normalized to the lowest β-actin value, and target protein levels were then normalized to their respective β-actin controls.
