# Supplementary figures and images for: Dedifferentiation-driven oncogenic stemness promotes tumor-sustaining adaptability in the intestinal epithelium
Source: Cell Death Dis. 2026 Apr 17;17(1):514. doi: 10.1038/s41419-026-08669-2 (PMC13216273; doi:10.1038/s41419-026-08669-2)

Figure S1

A

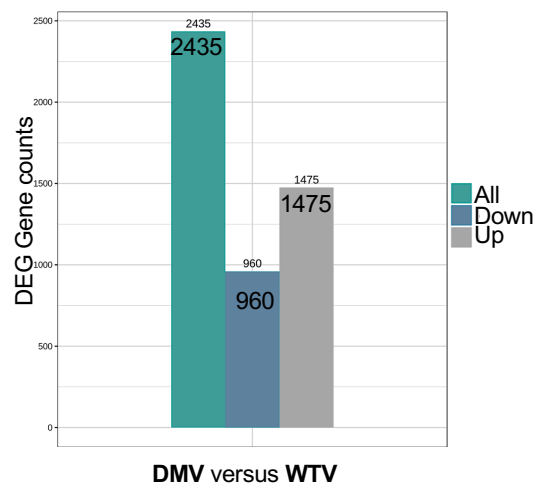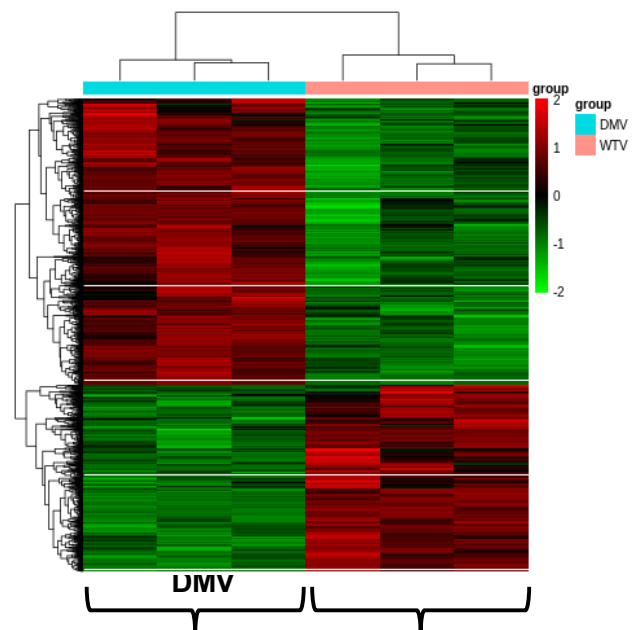

B

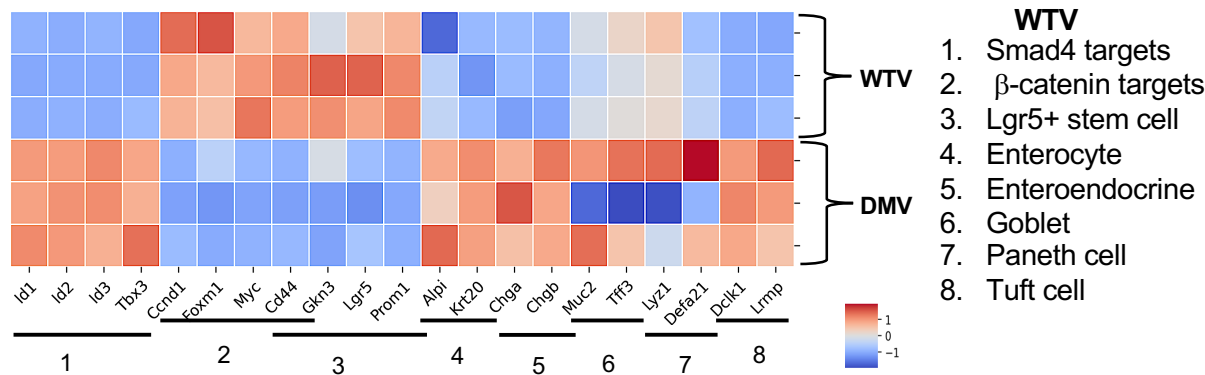

Supplement: Supplementary file 2 — Supplementary Figure 1 [file 41419_2026_8669_MOESM2_ESM.pdf]

Figure S2

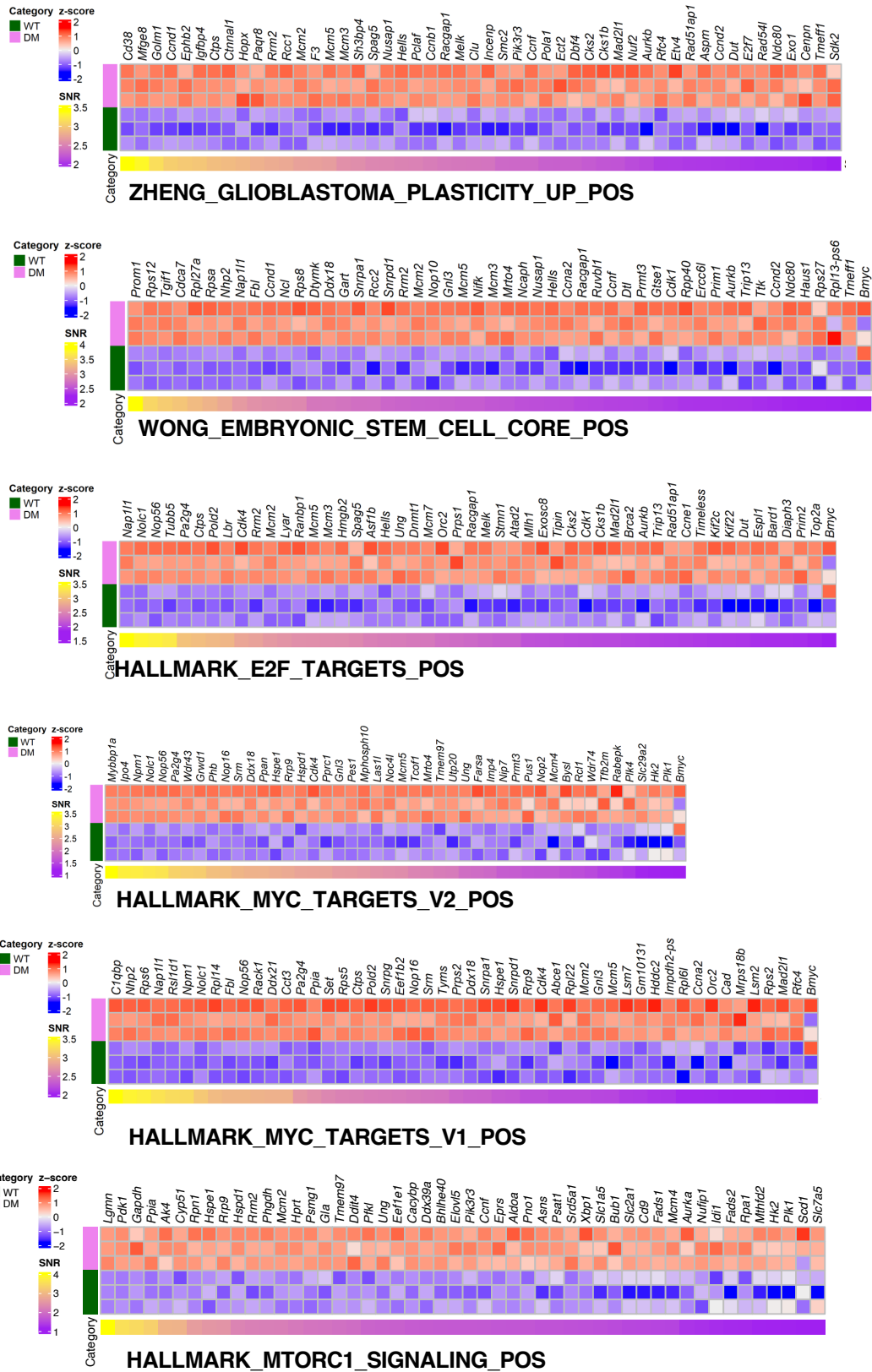

Supplement: Supplementary file 3 — Supplementary Figure 2 [file 41419_2026_8669_MOESM3_ESM.pdf]

**Figure S3**

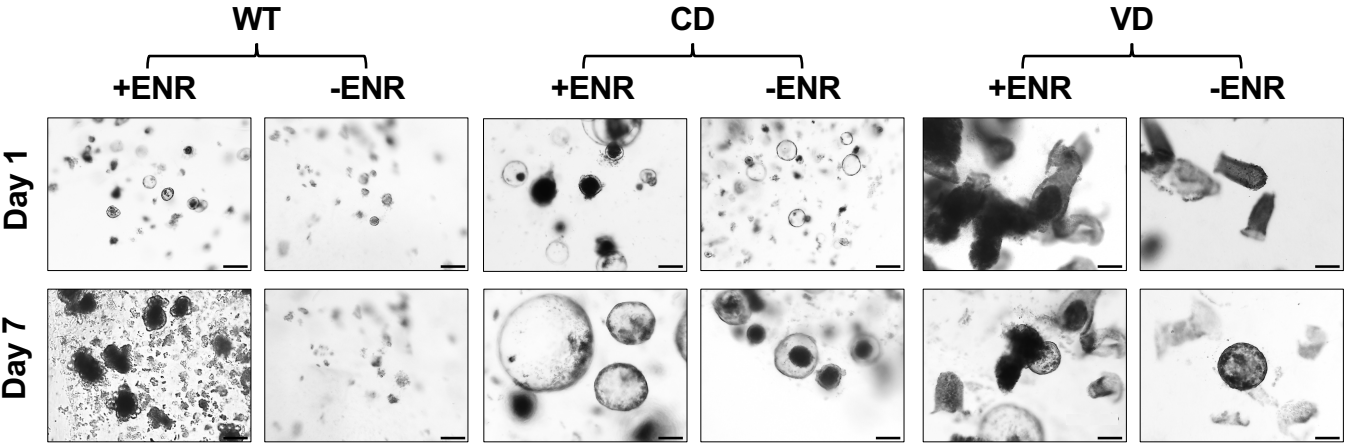

Supplement: Supplementary file 4 — Supplementary Figure 3 [file 41419_2026_8669_MOESM4_ESM.pdf]

Figure S4

Unfolded Protein Response

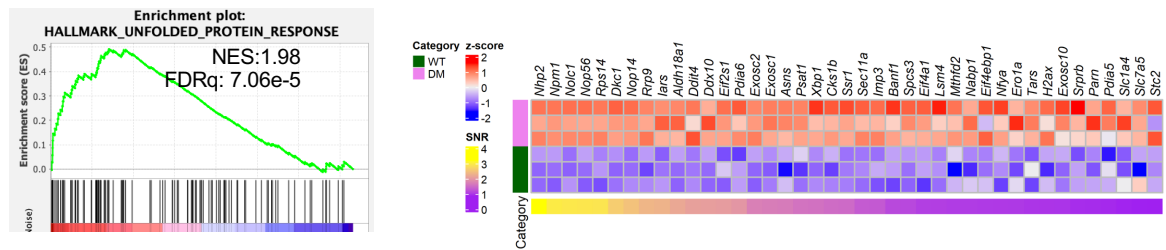

Supplement: Supplementary file 5 — Supplementary Figure 4 [file 41419_2026_8669_MOESM5_ESM.pdf]

Figure S5

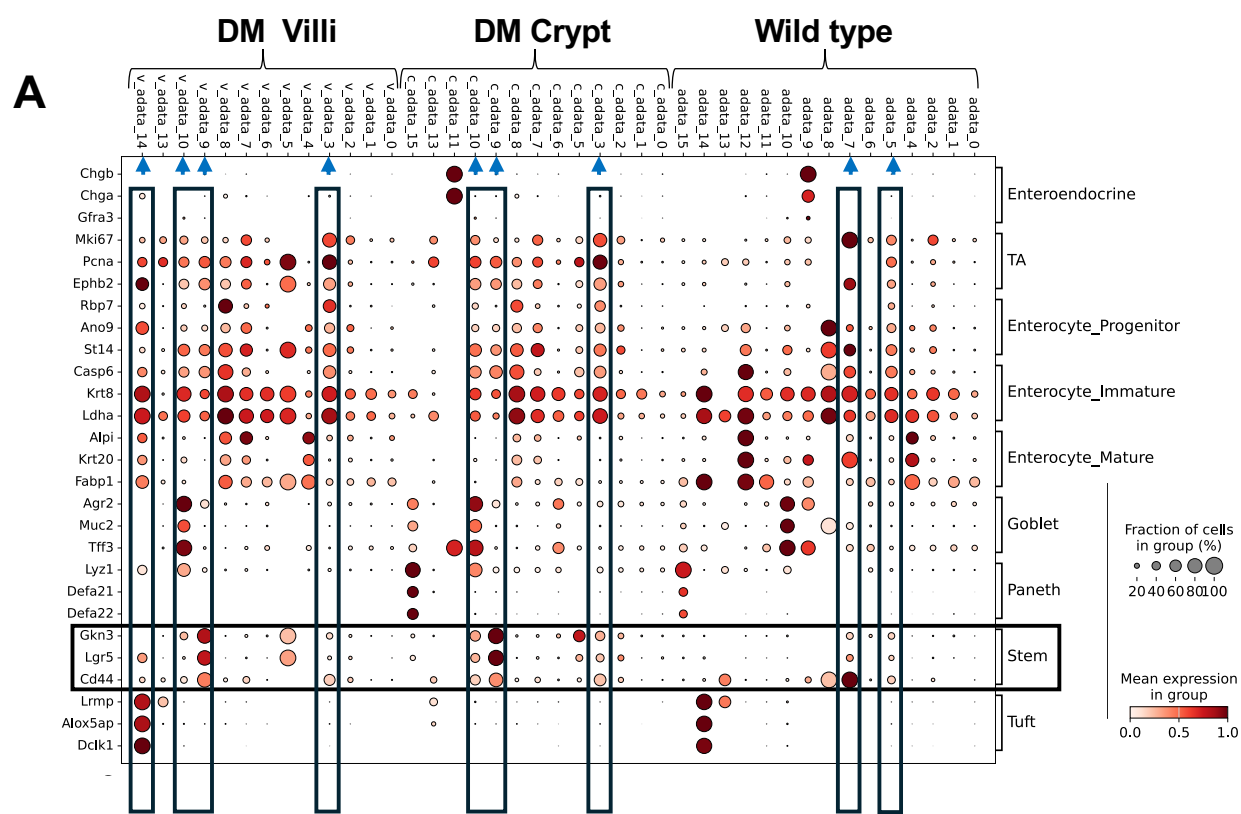

**B**

| c #    | Wild type     | DM villi | DM crypt |
|--------|---------------|----------|----------|
| leiden | control_adata | v_adata  | c_adata  |
| 0      | 3193          | 375      | 388      |
| 1      | 72            | 216      | 2556     |
| 2      | 1987          | 231      | 261      |
| 3      | 0             | 1046     | 1287     |
| 4      | 2002          | 37       | 0        |
| 5      | 1866          | 1        | 6        |
| 6      | 17            | 207      | 1632     |
| 7      | 3             | 1229     | 227      |
| 8      | 1             | 480      | 536      |
| 9      | 7             | 76       | 688      |
| 10     | 172           | 106      | 242      |
| 11     | 497           | 0        | 1        |
| 12     | 410           | 0        | 0        |
| 13     | 3             | 135      | 23       |
| 14     | 153           | 4        | 0        |
| 15     | 20            | 0        | 13       |
| Total  | 10403         | 4143     | 7860     |

Supplement: Supplementary file 6 — Supplementary Figure 5 [file 41419_2026_8669_MOESM6_ESM.pdf]

Figure S6

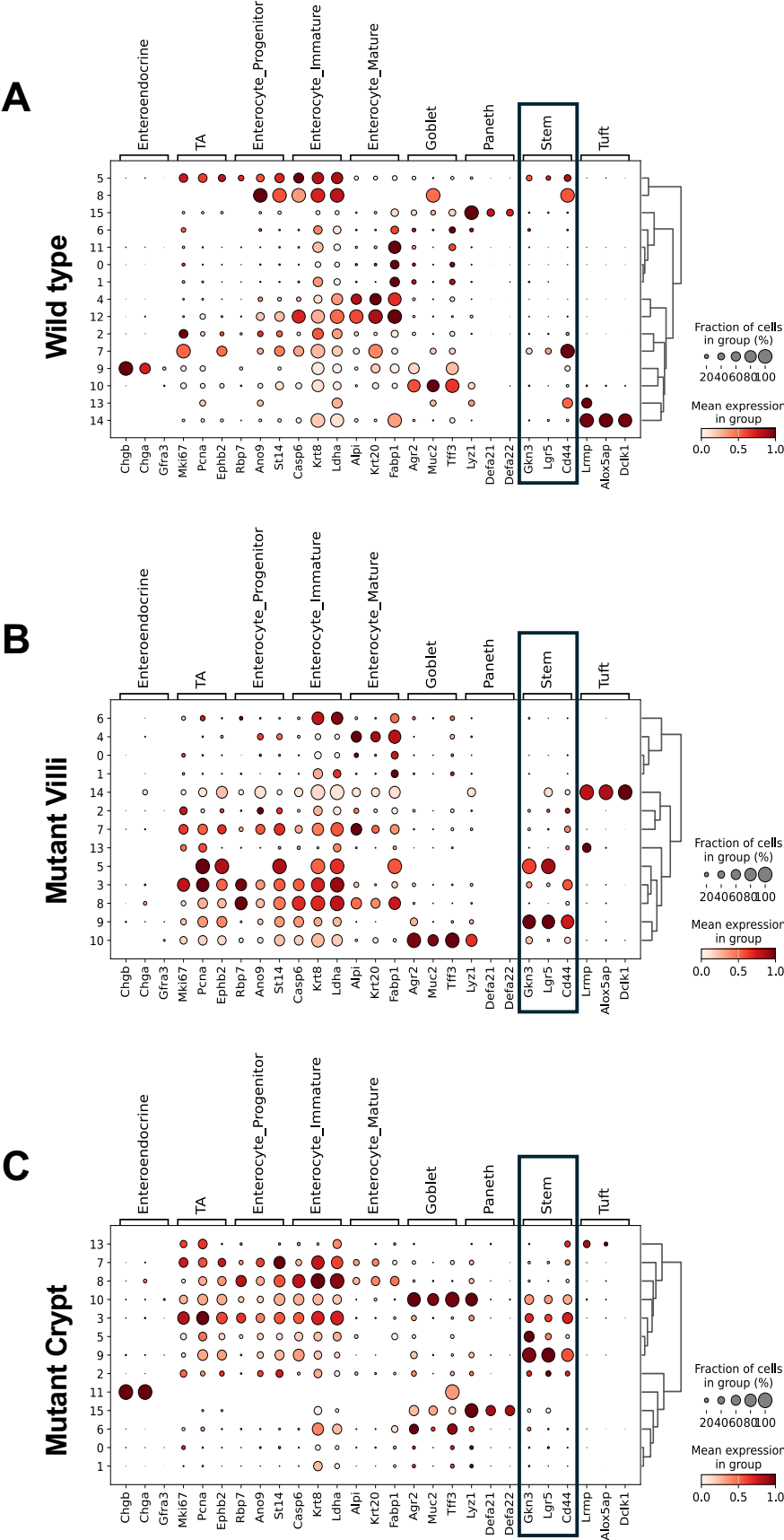

Supplement: Supplementary file 7 — Supplementary Figure 6 [file 41419_2026_8669_MOESM7_ESM.pdf]
