# Supplementary material for: Dedifferentiation-driven oncogenic stemness promotes tumor-sustaining adaptability in the intestinal epithelium
Source: Cell Death Dis. 2026 Apr 17;17(1):514. doi: 10.1038/s41419-026-08669-2 (PMC13216273; doi:10.1038/s41419-026-08669-2)
Supplement: Supplementary file 9 — Supplementary Table 2 [file 41419_2026_8669_MOESM9_ESM.docx]

Supplementary Table 2. Primary and secondary antibodies used for immunohistochemistry.

| **Antibody** | **Dilution** | **Catalog#** | **Company** |
| --- | --- | --- | --- |
| Anti-CD44 Rat mAb, clone IM7 | 1:1000 | 103002 | BioLegend |
| Anti-GLS, clone 1D16 | 1:500 | ZRB1798 | EMD Millipore |
| Cdx2 (D11D10) Rabbit mAb | 1:1000 | 12306S | Cell Signaling Technology |
| Cleaved Notch1 (D3B8) Rabbit mAb | 1:500 | Val1744 | Cell Signaling Technology |
| Gfp (D5.1) XP® Rabbit mAb | 1:100 | 2956S | Cell Signaling Technology |
| Human/Mouse EphB2 Antibody | 1:100 | AF467-SP | R&D Systems |
| Keratin 20 (D9Z1Z) XP® Rabbit mAb | 1:1000 | 13063S | Cell Signaling Technology |
| Lysozyme, Polyclonal, Unconjugated, Ig fraction | 1:2000 | A009902-02 | Agilent Technologies |
| Smad4 (D3R4N) XP® Rabbit mAb | 1:250 | 46535T | Cell Signaling Technology |
| Tom20 (D8T4N) Rabbit mAb | 1:200 | 42406S | Cell Signaling Technology |
| Goat anti-mouse IgG (H+L), Biotinylated | 1:300 | BA-9200 | Vector Laboratories |
| Goat anti-rabbit IgG (H+L), Biotinylated | 1:700 | BA-1000 | Vector Laboratories |
| Goat anti-rat IgG (H+L), Biotinylated | 1:300 | BA-9400 | Vector Laboratories |
