# Supplementary material for: Dedifferentiation-driven oncogenic stemness promotes tumor-sustaining adaptability in the intestinal epithelium
Source: Cell Death Dis. 2026 Apr 17;17(1):514. doi: 10.1038/s41419-026-08669-2 (PMC13216273; doi:10.1038/s41419-026-08669-2)
Supplement: Supplementary file 10 — Supplementary Table 3 [file 41419_2026_8669_MOESM10_ESM.docx]

Supplementary table 3. Primary and secondary antibodies used for western blot.

| **Antibody** | **Dilution** | **Catalog#** | **Company** |
| --- | --- | --- | --- |
| beta Actin Antibody (C4) | 1:1000 | sc-47778 | Santa Cruz Biotechnology |
| Pink1 Antibody (38CT20.8.5) | 1:1000 | sc-517353 | Santa Cruz Biotechnology |
| PRDX3 Rabbit PolyAb | 1:2000 | 10664-1-AP | Proteintech |
| PRDX6 Rabbit PolyAb | 1:2000 | 13585-1-AP | Proteintech |
| m-IgGk BP-HRP | 1:2000 | sc-516102 | Santa Cruz Biotechnology |
| mouse anti-rabbit IgG-HRP | 1:2000 | sc-2357 | Santa Cruz Biotechnology |
