# Supplementary material for: Dedifferentiation-driven oncogenic stemness promotes tumor-sustaining adaptability in the intestinal epithelium
Source: Cell Death Dis. 2026 Apr 17;17(1):514. doi: 10.1038/s41419-026-08669-2 (PMC13216273; doi:10.1038/s41419-026-08669-2)
Supplement: Supplementary file 11 — Supplementary Table 4 [file 41419_2026_8669_MOESM11_ESM.docx]

Supplementary Table 4. Materials used for single-cell RNA-sequencing sample preparation.

| **Product** | **Catalog#** | **Company** |
| --- | --- | --- |
| 40-micron filter | 542040 | Greiner Bio-One |
| 6-well TC plate | 10861-554 | VWR |
| 70-micron filter | 431751 | Corning |
| Bovine SerumAlbumin | 5217 | Tocris Bioscience |
| DAPI | 5087410001 | Sigma-Aldrich |
| Dead Cell Removal Kit | 130-090-101 | Miltenyi Biotec |
| Dispase | 7913 | STEMCELL Technologies |
| DNase | 7900 | STEMCELL Technologies |
| EDTA | 324504 | EMD Millipore |
| FITC anti-mouse/human CD44 [IM7] | 103006 | BioLegend |
| PBS | BP399-4 | Fisher Scientific |
