# Supplementary material for: Dedifferentiation-driven oncogenic stemness promotes tumor-sustaining adaptability in the intestinal epithelium
Source: Cell Death Dis. 2026 Apr 17;17(1):514. doi: 10.1038/s41419-026-08669-2 (PMC13216273; doi:10.1038/s41419-026-08669-2)
Supplement: Supplementary file 12 — Supplementary Table 5 [file 41419_2026_8669_MOESM12_ESM.docx]

Supplementary Table 5. Materials used for organoid preparation.

| **Product** | **Catalog#** | **Company** |
| --- | --- | --- |
| Advanced DMEM/F-12 | 12634010 | Thermo Fisher Scientific |
| Animal-Free Recombinant Murine EGF | AF-315-09 | PeproTech |
| B27 Supplement w/o Vit A (50x) | 12587010 | Thermo Fisher Scientific |
| Corning® Matrigel® Basement Membrane Matrix, Phenol Red-Free, *LDEV-Free | 356237 | Discovery Labware |
| Glutmax 1, 100x | 35050061 | Thermo Fisher Scientific |
| HEPES | 15630080 | Thermo Fisher Scientific |
| N2 Supplement | 17502048 | Thermo Fisher Scientific |
| N-Acetyl-L-cysteine,cell culture tested, BioReagent | A9165 | Sigma-Aldrich |
| Penicillin Streptomycin sol | 97063-708 | VWR |
| Recombinant Human R-Spondin 1 Protein | 4645-RS-025 | R&D Systems |
| Recombinant Murine Noggin | 250-38 | PeproTech |
