# Supplementary material for: Dedifferentiation-driven oncogenic stemness promotes tumor-sustaining adaptability in the intestinal epithelium
Source: Cell Death Dis. 2026 Apr 17;17(1):514. doi: 10.1038/s41419-026-08669-2 (PMC13216273; doi:10.1038/s41419-026-08669-2)
Supplement: Supplementary file 13 — Supplementary Table 6 [file 41419_2026_8669_MOESM13_ESM.docx]

Supplementary Table 6. Other reagents and Materials.

| **Product Name** | **Catalog #** | **Company** |
| --- | --- | --- |
| ABC-HRP Vectastain kit | PK-4000 | Vector Laboratories |
| BCA Protein Assay Kit | 0023224 | Fisher Scientific |
| Hematoxylin | 26030-20 | Electron Microscopy Sciences |
| Hypoxyprobe Red549 Kit | 701 | Hypoxyprobe, Inc. |
| ImmPACT (TM) DAB HRP Substrate | SK-4105 | Vector Laboratories |
| Methyl green | ZH0804 | Vector Laboratories |
| NaCl | S271-1 | Fisher Scientific |
| NaF | A13019-30 | Alfa Aesar |
| Paraformaldehyde | 15714-S | Fisher Scientific |
| PMSF | P7626 | Sigma-Aldrich |
| Proteases inhibitor | P8340 | Sigma-Aldrich |
| Sodium Vanadate | 72060 | Sigma-Aldrich |
| Tamoxifen | T5648 | Sigma-Aldrich |
| Triton x-100 | 0694 | VWR |
| TRIzol | 15-596-018 | Thermo Fisher Scientific |
